# Cytochemical identification of endocrine thymus of chicken in relation to aging 

**Published:** 2013

**Authors:** Uma Kanta Mishra

**Affiliations:** *Department of Anatomy, Histology and Embryology, Faculty of Veterinary Science and Animal Husbandry, Orissa University of Agriculture and Technology, Bhubaneswar, India.*

**Keywords:** Aging, Chicken. Endocrine cells, Serotonin cells, Thymus

## Abstract

Age related cytochemical changes of thymic endocrine cells were studied in 78 day old chicks at five day interval to age of day 60 employing a panel of cytochemical stains. Methenamine silver revealed cell morphology including cell processes distinctly while diamine silver revealed a stronger argentaffinity in these cells. The cells had greater affinity for diamine silver compared to methenamine silver (Argentaffin), followed by formaldehyde induced autofluorescence, argyrophilia, lead hematoxylin and HCl-toluidine. The chromaffin reaction was the weakest. Cytochemically, three different endocrine cell populations i.e. argentaffin cells, argyrophilic cells and amine precursor uptake and decarboxylation series (APUD)/chromaffin cells, formed the resident population of thymic endocrine cells. Occurrence of numerous serotonin storing cells, moderately frequent APUD cells, and fewer chromaffin as well as mast cells suggests for a conspicuous reservoir of amine storing cells in thymus. Morphologically argentaffin cells were of four types i.e. the peripherally granulated spherical cells (Type-I), densely granulated oval cells (Type-II), pyramidal argentaffin cells (Type-III) and diffusely granulated elongated cells (Type-IV). The type-II argentaffin cells were most frequent in the medulla followed by the type-I cells and the type-III cells. The type-IV cells were least in frequency. The age related changes in frequency of these cells are also discussed.

## Introduction

The endocrine nature of the thymus of chicken has been challenged several times in the past and requires morphological, cytochemical and functional authentications.^1^^,^^2^ With the advent of immunocytochemical techniques, cellular localization of few peptides like somatostatin, neurotensin, vasoactive intestinal polypeptide, serotonin and parvalbumin in thymus of chicken has been achieved.^3^^-^^5^ These chemical mediators are known hormones/neuro-secretions seen elsewhere in the body, for example in the mucosa of gut,^6^^,^^7^ respiratory tract,^8^ urogenital tract,^9^ and in brain.^10^ The endocrine nature of the thymic peptide/amine secreting cells needs deconvolution. The endodermal cells of pharyngeal pouch established the epithelial cells in thymus during embryonic life of chicks. Lymphocytes then invaded these epithelial cell clusters to establish the typical lympho-epithelial nature of the gland. Surprisingly, morphology of most endocrine cells of body resembles to that of the epithelial cells carrying ample secretion granules in their cytoplasm.^11^ The differential cytochemical behaviour of thymic endocrine cells in relation to aging of the chicks has not yet been worked out to the full extent though a number of cytochemical stains appear in the literature for demonstration of secretion granules of endocrine cells in general.^12^ In this study efforts were made to elucidate frequency distribution, morphology and histochemical properties of thymic endocrine cells of chicken during early post hatch period employing a panel of cytochemical stains. 

## Materials and Methods

Seventy eight day old apparently healthy white Leghorn male chicks (Deklab strain) were procured from Post Graduate Department of Poultry Science, Faculty of Veterinary Science and Animal Husbandry, Orissa University of Agriculture and Technology, Bhubaneswar, India. The chicks were raised up to 60 days of age under ideal and uniform husbandry conditions in Department of Anatomy and Histology on deep litter system. The birds were fed with broiler starter and grower rations at recommended ages. The chicks were vaccinated against Newcastle disease (NCD) at age of day 5 at a dose rate of 0.03 mL per chick intranasally using NCD Virus (NCDV, Lasota, lentogenic strain, Venky India) and the booster dose was repeated at age of day 21. They were also vaccinated against infectious bursal disease (IBD) at age day 14 at a dose rate of 0.03 mL per chick intranasally using IBD virus (IBDV, Intermediate strain, Venky, Hyderabad, India). The booster dose was repeated at age of day 35. A five day interval study was conducted on six birds (in each age group) selected randomly from the pen, to record the histomorphological and cytochemical features, and frequency distribution of the thymic endocrine cells. On the day of observation the chicks were euthanized by cervical dis location according to Ethical Committee of the author’s institute. The cervical and thoracic lobes of thymi were then dissected out using hand lens and fixed in 10% buffered neutral formalin for routine preparation of 5-6 µm thick serial paraffin sections. The sections were subjected to following panel of cytochemical reactions: (i) Masson-Hamperl’s (MH) silver reaction using methenamine silver (MH-MS) as well as diamine silver (MH-DS) for argentaffin cells; (ii) Grimelius silver (GS) for argyrophilic cells;^13^ (iii) HCl-Toluidine blue (HTB) stain for peptide secreting cells;^14^ (iv) Lead-Haematoxylin (LH) stain for APUD cells;^15^ (v) Chromaffin reaction using acidified potassium dichromate and potassium chromate (PDPC), (pH = 5) as well as potassium iodate (PI) reaction, with and without hematoxylin as counter stain, on frozen sections, for the chromaffin cells; (vi) Formaldehyde induced autofluorescence (FAF) technique for serotonin containing cells,^16^ where the cells were examined under a fluorescent microscope using blue filter; (vii) Ninhydrin-Schiff (NS) reaction for protein;^12^ and (viii) Toluidine blue (TB) for mast cells.^13^ A separate stain for mast cells was employed as they are known to contain biogenic amines that are also present in the endocrine cells for differential study. The morphology and distribution of endocrine cells were documented using a research microscope (Model DM 2500, equipped with camera model DFC290, Leica Microsystems, Wetzlar, Germany) with fluorescent attachment. The frequency distribution of cells was estimated by counting cells in every fifth field of tissue section randomly. From the preliminary study it was seen that the number of cells discerned per field varied from 1 to 5 cells and rarely more than five cells. Therefore, the numbers were categorized into different frequency groups such as sparse/occasional (±) with 1 cell per field, few (+) with 2 cells per field, moderate (++) with 3 cells per field, abundant (+++) with 4 cells per field, and superfluous (++++) with more than 4 cells per field. The age related changes in frequency distribution of endocrine cells were recorded as increase and decrease in cell number. 

## Results

**Fig. 1:**
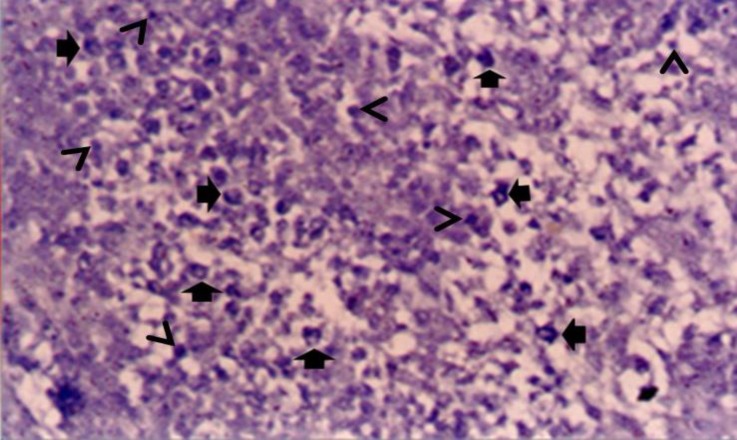
Cytochemical reactions of endocrine cells of thymus of chicken at different post hatch ages; Diffuse scatter of cells of amine precursor uptake and decarboxylation (APUD) series in medulla at day 30 (arrow). Note the small large lymphocytes (arrow head), (Lead-Haematoxylin, 400×).

**Fig. 2:**
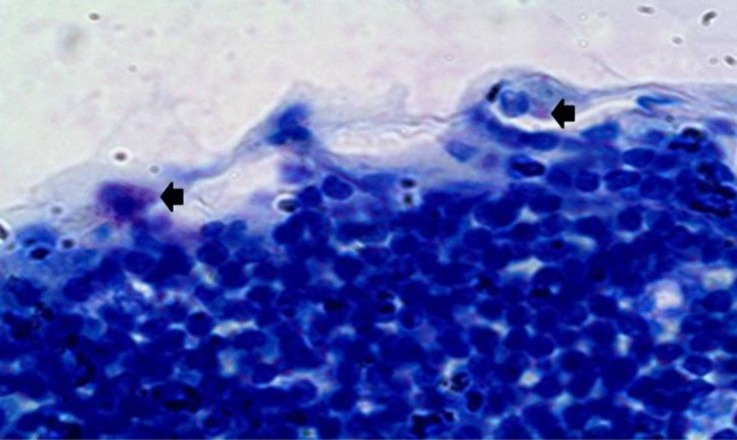
Mast the cells in capsule at day 10 (arrow). The cortical lymphocytes stained dense blue, (Toluidine blue, 1000×).

**Fig. 3:**
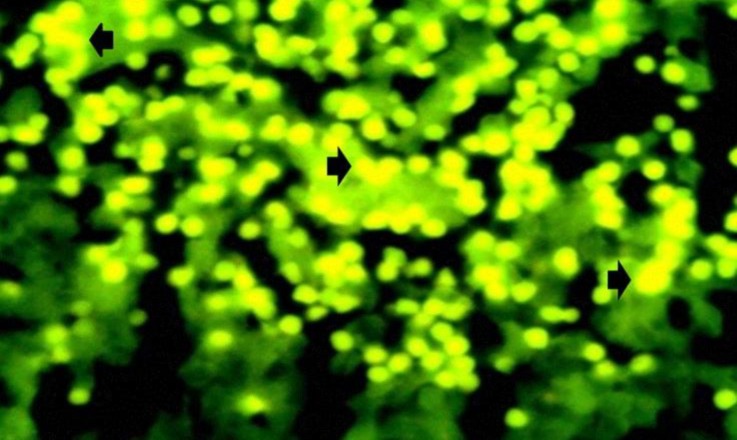
Numerous formaldehyde induced auto fluorescent serotonin storing cells in medulla (arrow) at day 50, (Formaldehyde induced auto fluorescence, 400×).

An important consideration was made on effect of age on the histomorphological characters, cytochemical behavior and frequency distribution of endocrine cells of thymic parenchyma of male chicken during early post hatch period at five days interval. A brief record on region specific distribution of the thymic endocrine cells of chicken at PD1 and their age related changes are summarized in Table 1. A moderate number of ovoid or polyhedral APUD cells, as revealed by LH stain, were diffusely scattered in the thymic medulla. A moderate number of such cells were also evident in the capsule, septa, pericapillary wall, medulla and cortico-medullary junction, and were few in the cortex. Cell frequency increased from moderate to abundance in the capsule and cortico-medullary junction on PD20 and in the medulla on PD25 (Fig. 1). The cortical cells increased from few to moderate by PD 30 (Table 1). The TB reactive mast cells were oval in shape with dense granulations and contained eccentric and euchromatic oval nuclei. Most of these cells were distributed in single but few were seen in small clusters. They were few in the central medulla and became moderate in number by PD25. Cells were particularly seen in close vicinity of capillaries or free RBCs. This peri-capillary cell distribution declined to few by PD25. Cells could not be seen in the cortices and were few in the capsule (Fig. 2) and interlobular septa. The thymic corpuscles revealed sparse cells (Table 1). Abundant formaldehyde induced auto-fluorescent serotonin storing cells were dispersed in the medulla as diffuse or discrete aggregations at age of day 1 and were distinctly polyhedral in shape. Isolated scatters of these cells in abundance were also evident at the cortico-medullary junction. The cells were few in the cortices. The cell frequency at pericapillary locations was sparse on PD1 and it increased to moderate by PD30. The cell number in the cortico-medullary junction and medulla increased to superfluous by PD10 and PD20, respectively (Table 1, Fig. 3). Few dark brown PDPC stained chromaffin cells were distributed in the medulla of thymi. The cells were sparse in capsule, septa, cortico-medullary junction and in wall of blood capillaries (Table 1). The frequency of these chromaffin cells did not change with age of the chicks. The chromaffin reaction was weak with PI where cells appeared light brown in color and the number of reactive cells was quite fewer. The cells contours were indistinct with both PDPC and PI reactions. 

**Fig. 4:**
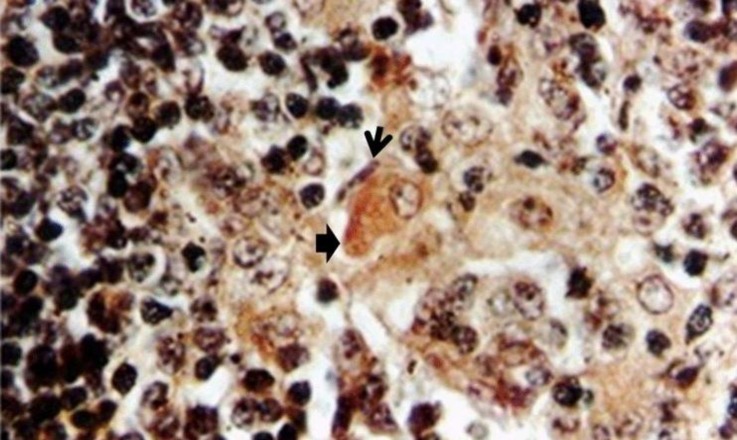
Argyrophilic cell with dark brown granular cytoplasm (solid arrow) in medulla adjacent to the capillary bed at day 40. Note capillary endothelial cell (line arrow), (Grimelius silver, 1000×).

Most of the cortical lymphocytes had a thin rim of blue cytoplasm with HTB stain for peptide secreting cells while the large ovoid or polyhedral cells with central euchromatic nuclei, were few in peripheral part of cortices (close to septa) and cortico-medullary junction. They were sparse in medulla, capillary wall and septa at PD1. The cell frequency became moderate in cortex by PD20, in medulla by PD35 and in cortico-medullary junction by PD40. Other thymic regions did not evince change in frequency of endocrine cells as the chicks aged (Table 1). Diffuse positive reactions for proteins were seen in throughout thymic parenchyma with NS except the cortical zone and cortico-medullary junction which had a slightly stronger reaction than the medulla. There was no noticeable change in the degree of NS reaction of thymus with advancing age in the chicks (Table 1). The GS stain elucidated few argyrophilic cells around blood capillaries (Fig. 4) and in the medulla of thymi. They were sparse in the cortico-medullary junction and could not be seen in other regions of thymus. The cells were polyhedral in shape with round vesicular eccentric nuclei and dark brown to light brown granular cytoplasm.

**Table 1:** Age related changes in frequency distribution of endocrine cells in thymus of chicken at post hatch day one.

**Thymic regions**	**Cytochemically reactive endocrine cells**							
	*1	2	3	4	5	6	7	8
**Capsule**	†-	-	-	++ ‡ (+++20)	±	-	+	+
**Cortex**	-	-	+ (++20)	+ (++30)	-	+	-	++
**Medulla**	+++	+	± (++35)	++ (+++25)	+	+++ (++++20)	+ (++25)	+
**Cortico-medullary junction**	+++	±	+ (++40)	++ (+++20)	±	+++ (++++10)	-	++
**Thymic corpuscles**	++	-	-	-	-	-	±	+
**Blood capillary wall**	++	+	±	++	±	± (++30)	++ (+25)	+
**Septa**	-	-	±	++	±	±	+	+

* Cytochemical reactions: 1. Argentaffin, 2. Argyrophil, 3. HCl Toluidine blue (peptide), 4. Lead Hematoxylin, 5. Chromaffin, 6. Formaldehyde induced auto fluorescence (serotonin), 7. Toluidine blue (mast cells), and 8. Ninhydrin Schiff (protein).

† Cell frequency: - (0 cells per field), ± (1 cell per field), + (2 cells per field), ++ (3 cells per field), +++ (4 cells per field) and ++++ (more than 4 cells per field).

‡ The symbols in parenthesis represent changes in frequency distribution of cells and the numbers in parenthesis represent the age of chicken in days.

**Fig. 5:**
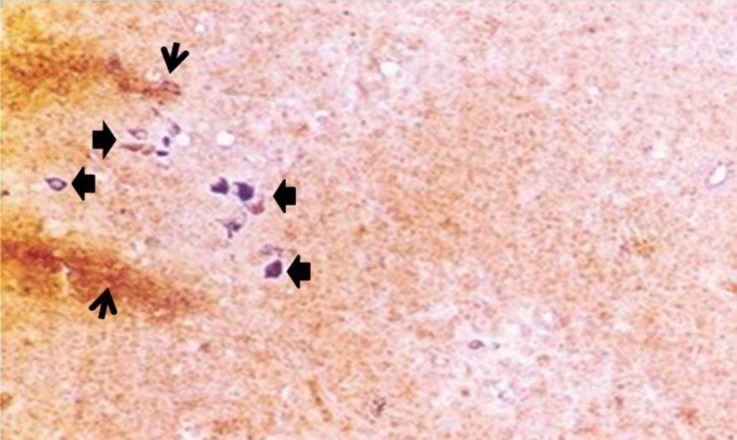
Distribution of argentaffin cells in medulla close to cortico-medullary junction (solid arrow) at day 5. Note the interlobular septa (line arrow), (Masson-Hamperl’s Diamine silver, 200×).

**Fig. 6:**
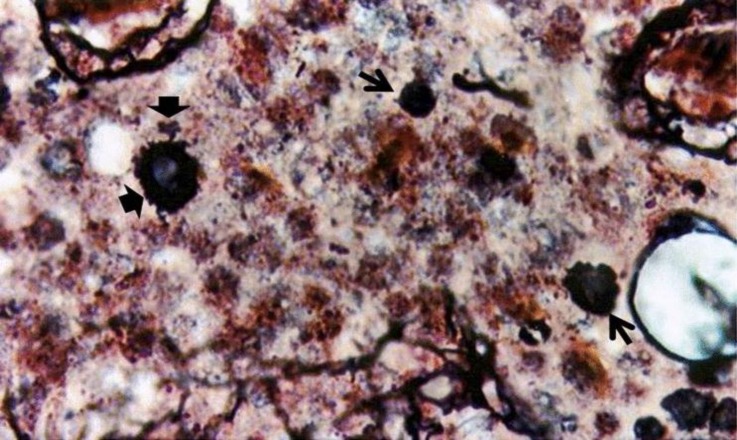
Argentaffin cells i.e. peripherally granulated spherical endocrine cells (type-I) with beaded cytoplasmic process (solid arrow), and small and large sized densely granulated oval endocrine cells (type-II) (line arrow) in perivesicular location at day 25. Some of the medullary reticular fibers also stained black, (Masson-Hamperl’s methenamine silver, 1000×).

**Fig. 7:**
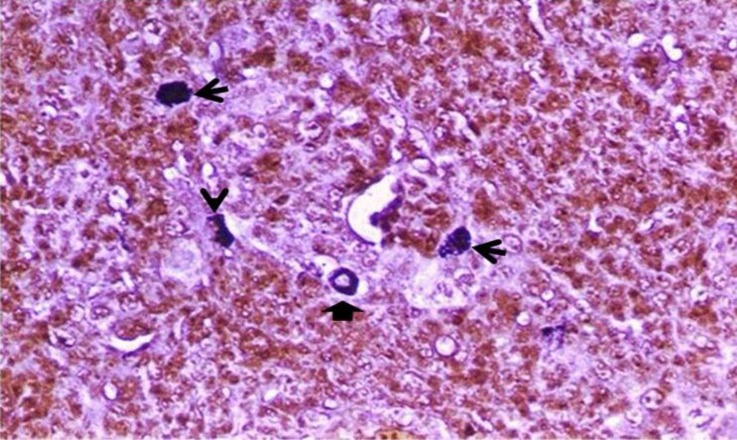
Histomorphology of argentaffin cell types i.e. peripherally granulated spherical cell (type-I) (solid arrow), densely granulated oval cell (type-II) (line arrow), and diffusely granulated elongated cells (type-IV) (arrow head) in medulla at day 35, (Masson-Hamperl’s methenamine silver, 400×).

These cells did not reveal age related changes in frequency distribution (Table 1). Several argentaffin cells were localized either in single or in small groups, i.e. 2-3 cells per group, frequently within the medulla and sometimes close to the cortico-medullary junction (Fig. 5). These cells were often seen in the vicinity of capillary wall, thymic vesicles and corpuscles of the medulla. The argentaffin cells were seen in black color by MH-DS technique. The cytoplasmic granulation pattern and the cell processes were not well defined with this technique. The MH-MS revealed the morphology of the argentaffin cells very distinctly with conspicuous localization of the intracytoplasmic secretion granules. The cell processes were also clearly discerned with this technique (Fig. 6). Morphologically four different kinds of argentaffin cells (MH-MS reactive) were identified. The peripherally granulated spherical cells (cell type-I), cells of this category were spherical in shape with centrally located vesicular and spherical nuclei. Occasionally some of these cells were expressing beaded cytoplasmic processes. The secretory granules were stained dense black and distributed throughout the cytoplasm around the nuclei giving the appearance of peripheral granulation (Figs. 6 and 7). A moderate number of cells appeared in central part of medulla and cortico-medullary junction at age of PD1. Such cells but with light diffuse scatter of intra-cytoplasmic secretory granulations were observed in moderate number within the thymic corpuscles by PD5. Cell frequency increased to abundance in medulla and cortico-medullary junction by PD15 (Fig. 5).

The densely granulated oval cells (cell type-II) were heavily studded with dense black stained secretory granules obscuring the nuclei. The cells occurred in two sizes i.e. small and large (Figs. 6 and 7). A moderate number of cells were seen in medulla, cortico-medullary junction and in vicinity of capillary wall on PD1. The medullary cell frequency increased rapidly with age and became abundant by PD25 (Fig. 6). The pyramidal argentaffin cells (cell type-III), cells were pyramidal or wedge shaped with round vesicular eccentric nuclei. The secretory granules were black to dark brown in color and distributed in the basal zone of the cell being infra nuclear in position. A moderate number of these cells appeared in the medulla at PD1 and they were sparse in the cortex. As the chicks grew older the cell frequency increased slowly and became abundance by PD40 in the medulla of thymi. The diffusely granulated elongated cells (cell type-IV), cell belonging to this category were few and elongated in outline. They revealed smallest amount of secretory granules in their cytoplasm which stained light brown to black with argentaffin reaction (Fig. 7). Frequently the cell contour could not be distinguished. A moderate number of cells appeared in the medulla up to 30 days of age and then followed a rapid decline. By PD40 cell frequency decreased to a small number. Irrespective of their type, argentaffin cells in general revealed abundance distribution in medulla and cortico-medullary junction, and moderate distribution in thymic corpuscles and pericapillary wall at PD1 (Table 1). At any given age of chicken during this study, the type-II argentaffin cells were most frequently seen within the thymic medulla followed by the type-I cells and the type-III cells. The type-IV cells were least in incidence.

## Discussion

A moderate frequency of LH reactive APUD cells had a constant and diffuse distribution within the medulla while they were not uncommon at other locations of the thymus of chicken in all the age groups studied except for thymic corpuscles where they were absent. The cells became abundant in cortico-medullary junction and capsule by PD20, in medulla by PD25 and in cortex by PD30. These cells are reported to decarboxylate the amine precursors into biogenic monoamines that are stored within the secretory granules. The carboxamido groups of the granule proteins reacted with the LH giving a bluish black colour to the cells.^15^ All the APUD cells which evince masked basophilia also revealed fluorescence metachromatia by acridine dyes.^15^^,^^17^ In thymic medulla of chicken two types of such monoamine containing endocrine cells were reported to appear.^3^ One, storing 5-hydroxytryptamine, gave positive argentaffin, chromaffin and ferric ferricyanide reduction reaction. The other one was devoid of 5-hydroxy-tryptamine but when supplied with L-dopa could decarboxylate it into dopamine, stored in cytoplasm. Both the cell types were positive to GS and HTB reaction suggesting presence of peptides with masked carboxyl groups and were sorted into family of monoamine secreting endocrine cells.^3^ The results of this study confirmed an age dependent rise in APUD cell frequency in medulla and cortico-medullary junction and thus acknowledged medulla to be a large reservoir of APUD cells in chicken. Further, it also affirms that large quantities of biogenic amines are supposedly available within the thymus. Mast cells stained metachromatically with toluidine blue.^16^ Most of the cells were densely granulated with euchromatic nuclei. Their frequency increased from few to moderate in medulla by PD25 and the reverse distribution was seen in peri-capillary locations at about same age. The cell frequency in other thymic regions did not reveal age related changes. They were reported to maintain a reverse relation between their number and blood basophiles in a given species i.e. mammals have numerous mast cells and few basophiles and this is reverse in birds. Mast cells were reported to appear first in the thymus of embryonic chicken on day 15 and by day 17 they are profuse and the number declined at hatching.^18^^,^^19^ Although in human thymus some of these cells were located close to hematopoietic foci, they were not associated with angiogenesis except in case of thymoma. One conclusion comes from the present study that like APUD cells, mast cell population could be another source of thymic monoamines which were seen to exert their action in a paracrine manner.^19^ The formaldehyde induced autofluorescent serotonin storing cells were abundant in the cortico-medullary junction and medulla at PD1 and became superfluous by PD 10 and 20, respectively. Their number increased from sparse to moderate in pericapillary locations by PD30 while rest of the thymic regions did not reveal age related changes. Such cells were distributed in the thymic medulla of rat and were suggested to constitute an integral component of all immunocompetent organs of the body.^20^^-^^22^ Present findings on the metachromatic mast cells, APUD and serotonin storing cells when compared with other reports lend support to the conclusion that biogenic monoamines stored in these cells might be the main chromogens for toluidine blue induced meta-chromatia as well as for the LH reactivity of the APUD cells.^3^^,^^15^^,^^17^ However the serotonin storing cells seemed to constitute a separate population of monoamine storing cells in thymus of chicken which emitted intense yellow fluorescence following exposure to formaldehyde vapors. Further, the constant occurrence of a population of moderate number of aminergic (APUD, serotonin and mast) cells at pericapillary locations might authenticate their hemocrine mode of functions**. **According to the results of this study, few dark brown chromaffin cells reactive to acidified chromium salts (PDPC) were seen in thymic medulla with sparse distribution at other locations. Chromaffin cells did not show any change in frequency as age of the chicks advanced. The PI reactive chromaffin cells were diffuse light brown in colour and constituted a small fraction of the total thymic chromaffin population. Thus, thymic chromaffin cells evinced stronger affinity for PDPC as compared to the PI. However chromaffin reaction failed to reveal a distinct cell contour. According to previous studies, the chromaffin cells of the adrenal medulla, gut mucosa and liver capsule yielded strong reaction with PI by forming complexes such as 2-iodoadrenochromes and 2-iodonoradrenochromes which appeared as dark brown to yellowish brown precipitates in tissue sections. Since 2-iodoadrenochrome was a water soluble complex, the reaction suitably demonstrated the noradrenaline cells.^16^^,^^23^ Hence, the probability that thymic chromaffin cells store noradrenaline cannot be ruled out. 

The lymphocytes had a feeble cytoplasmic reaction with HTB. Although the large ovoid or polyhedral peptide secreting cells could be identified using HTB, their affinity to toluidine blue seemed to be moderate as compared to their affinity for silver salts. The Cell frequency increased from few to moderate by PD20 in the cortex and by PD40 in the cortico-medullary junction while the sparse cells of medulla increased to moderate by PD35. Occurrence of these cells in thymus up to age of PD60 argues for requirement of these cells as they were reported to liberate the peptide hormones.^5^^,^^15^ A moderate quantity of the proteins could be localized in cells of cortex and cortico-medullary junction while in other regions they were few giving the light and diffuse reaction. The cortices evinced slightly stronger NS reaction than the medulla and this might account for the densely packed cortical lymphocytes. The stain was reported to elucidate the structural proteins,^12^ and in the current context they were likely to be involved in thymic cell synthesis. Few polyhedral argyrophilic cells were dispersed in the medulla up to 60 days of age, often adjacent to capillary wall. The cell frequency did not change as the chicks aged. The cytoplasm was marked by light to dark brown granulation. Their proximity with the capillary endothelium supported for endocrine function of the cells. 

The argentaffin cells outnumbered the argyrophilic cells. The age dependent increased frequency of the argentaffin cells particularly within the medulla was associated with vesicles, capillaries, corpuscles and epithelial cells. Often some of them revealed beaded cytoplasmic processes. With MH-MS the argentaffin cells appeared to be pleomorphic and elucidated distinct distribution pattern of secretory granules in the cytoplasm including the cell processes. This allowed a fair classification of these argento-reducers into four types i.e., the peripherally granulated spherical cells (Type-I) whose frequency increased from moderate to abundance by PD15 in medulla and cortico-medullary junction, the densely granulated oval cells (Type-II) revealed increase in frequency from moderate to abundance in medulla by PD25, the pyramidal argentaffin cells (Type-III) became abundant by PD40 whereas they were moderate at PD1 in the medulla and the diffusely granulated elongated cells (Type-IV) were moderate in number in medulla up to PD30 and therefrom declined to few by PD40. Such a classification would help us to assess the cell type(s) which get affected in a particular thymic disorder. So far, no attempts have been made to put forth such nomenclature of thymic endocrine cells on morphological and cyto-chemical basis. Though, immunocytochemical classifications depending on cellular localization of specific peptides/ amines, reveal several cell categories. (i) Two cell-types: cells immunoreactive to neurotensin and somatostatin, were few at hatching but proliferated rapidly during first week of age in chicken. The neurotensin cells were GS positive and Hellerström-Hellman silver negative while somatostatin cells were GS negative and Hellerström-Hellman positive. Both cell types were non-argentaffin by nature.^24^ (ii) Four cell-types: cells immunoreactive to M-enkephalin, neuropeptide Y, substance P and vasoactive intestinal polypeptide in thymic medulla of chicken.^5^ Previously the neuron specific enolase and chromogranin, the known markers of diffuse neuro-endocrine system, are localized within the electron dense secretion granules of thymic medullary cells.^25^ The chromogranin-A immunoreactive cells of medulla were located close to cortico-medullary junction. They were mostly small and round or oval in shape while few were large and had conspicuous cell processes which mediated paracrine functions.^26^ Thus, cells are reported to give neuron specific enolase, somatostatin and chromogranin immunoreactivities. In consonance with these reports the present observations on cytochemistry of thymic endocrine cells clearly supported the idea that “if not all, at least a part of the thymic endocrine cells in chicken empirically belong to the ‘diffuse neuro-endocrine system”.

In conclusion, this study revealed methenamine silver to be the stain of choice for precise histochemical identification of thymic endocrine cell morphology, cytoplasmic granulation pattern and cell processes while diamine silver demonstrated comparatively a strong degree of argentaffinity in these cells. The tinctorial affinity of thymic endocrine cells was maximum for the silver salts i.e. more affinity to diamine silver as compared to methenamine silver, followed by formaldehyde induced autofluorescence, Grimelius argyrophilia, Lead hematoxylin and HCl-toluidine blue reaction. The chromaffin reaction was the weakest one. Cytochemically, at least three different endocrine cell populations, namely, the argentaffin cells, argyrophilic cells and the APUD/chromaffin cells, constituted a resident endocrine cell population in thymus of chicken. The appearance of moderate number of APUD cells, numerous serotonin storing cells and fewer chromaffin cells and mast cells suggested that there was a conspicuous reservoir of amine storing cells in thymus of chicken. Morphologically four types of argentaffin cells i.e. peripherally granulated spherical cells (Type-I), densely granulated oval cells (Type-II), pyramidal argentaffin cells (Type-III) and diffusely granulated elongated cells (Type-IV) were present in thymus of chicken. The type-II argentaffin cells were most frequent within the thymic medulla followed by the type-I and the type-III cells. The type-IV cells were least in frequency. As age of the chicks advanced, cells of type-I and II increased rapidly in frequency and cells of type-III revealed a slight increase while frequency of cell type-IV declined after 30 days of age. 
